# Alterations in subgenual anterior cingulate cortex functional connectivity underlie depressive symptoms in chronic insomnia disorder

**DOI:** 10.3389/fpsyt.2026.1765885

**Published:** 2026-02-11

**Authors:** Yuan He, Shaoxiang Zhong, Duan Liu, Haoyu Li, Junyao Wang, Yanling Chen, Ronghua Xu, Chunhua Xi, Liang Gong

**Affiliations:** 1 North Sichuan Medical College, Department of Neurology, West China School of Medicine, Sichuan University, Sichuan University affiliated Chengdu Second People’s Hospital, Nanchong, Sichuan, China; 2 Department of Neurology, The affiliated Hospital of Southwest Medical University, Chengdu Second People’s Hospital, Luzhou, Sichuan, China; 3 Department of Neurology,West China School of Medicine, Sichuan University, Sichuan University affiliated Chengdu Second People’s Hospital, Sichuan Provincial Engineering Research Center of Brain-macine Interactive Neurodulation, Chengdu, Sichuan, China; 4 Chengdu Medical College, Department of Neurology, Chengdu Second People’s Hospital, Chengdu, Sichuan, China; 5 Department of Neurology, The Third Affiliated Hospital of Anhui Medical University, Hefei, Anhui, China

**Keywords:** depression, functional connectivity, insomnia, resting-state functional magnetic resonance imaging, subgenual anterior cingulate cortex

## Abstract

**Background:**

Chronic insomnia disorder (CID) and depression exhibit high comorbidity, yet the underlying neurobiological mechanisms remain poorly understood. Neuroimaging meta-analyses suggest the subgenual anterior cingulate cortex (sgACC) is a key node, but the characteristics of its network connectivity in CID patients with depressive symptoms (CID-D) are unclear.

**Methods:**

This study enrolled 197 participants: 66 CID patients without depression (CID-nD), 67 CID-D patients, and 64 good sleep controls (GSC). Using resting-state functional magnetic resonance imaging (fMRI), we compared sgACC-based functional connectivity (FC) across groups. We also examined correlations between altered FC and clinical symptoms, and investigated whether altered sgACC FC mediated the relationship between insomnia severity and depressive symptoms.

**Results:**

Significant group differences in sgACC FC were found in the left inferior temporal gyrus (ITG), inferior frontal gyrus (IFGtri), right supplementary motor area (SMA), postcentral gyrus (POCG), and medial superior frontal gyrus (SFGmed). Specifically, compared to CID-nD, CID-D patients showed increased FC with ITG.L and IFGtri.L, and decreased FC with SMA.R and POCG.R. FC between sgACC and ITG.L or IFGtri.L was positively correlated with depressive symptoms, while sgACC-POCG.R FC was negatively correlated. Mediation analysis revealed that sgACC-ITG.L FC partially mediated the link between insomnia and depressive symptoms.

**Conclusion:**

Our findings identify specific alterations in sgACC functional network in CID patients with comorbid depression. The mediating role of sgACC-ITG.L connectivity highlights a potential neural pathway through which insomnia contributes to depressive symptoms, identifying a putative target for neuromodulation therapies.

## 1 Introduction

Chronic insomnia disorder (CID) is characterized by persistent difficulty initiating or maintaining sleep, with daytime impairment persisting for at least four weeks (1). This prevalent sleep disorder affects 4-20% of the global population and is associated with adverse physical health outcomes like cardiovascular issues (2), obesity (3, 4), and heightened susceptibility to diabetes (5), as well as negative impacts on mental health, such as depression (6, 7), anxiety disorders (8) and post-traumatic stress disorder and anxiety (9). Mood disorders, particularly major depressive disorder (MDD), which has a prevalence of 5-10% (10), has been linked to insomnia, with studies indicating that individuals with insomnia are three to four times more prone to developing major depressive disorder compared to the general population (11, 12), while about two-thirds of individuals with depression also have insomnia (13). Previous work indicates that the two conditions frequently co-occur and interact in the pathogenesis of the disease (14, 15). However, the neurobiological mechanisms underlying the comorbidity of insomnia with depressive symptoms remain unclear.

The advancement of neuroimaging technology has facilitated numerous investigations into the neurobiological underpinnings of CID. Prior research has identified gray matter atrophy in regions such as the precuneus, parietal cortex, prefrontal cortex, superior temporal gyrus, hippocampus and the Orbitofrontal cortex (OFC) among individuals with insomnia (16). In the resting-state functional connectivity study, significant changes were found in functional connectivity of several key brain networks. For example, functional connections within the default mode network (DMN), particularly between the prefrontal cortex (PFC) and the anterior cingulate cortex (ACC) and posterior cingulate cortex (PCC) (17–19), are reduced. At the same time, functional connections within the salience network (SN), especially among the insular lobes, amygdala and ACC, were enhanced (20). Furthermore, functional connectivity within the prefrontal parietal lobe or executive control network (FPN or ECN) was weakened (21), whereas that of the sensorimotor network (SMN) was increased, especially in the precentral gyrus and other sensorimotor regions (22). Functional connectivity within the dorsal attention network (DAN) increased (22). Although a large number of studies have demonstrated characteristic changes in brain network connections in CID patients, the results are not uniform, and depression associated with anxiety is one of the reasons for the difference in results. In CID patient with depressive symptoms, a decrease in gray matter volume in OFC was found to correlate with severity of depressive symptoms in terms of brain structure (23). There were also significant changes in several key brain networks, including the reward network, SN, DMN, FPN, SMN, with stronger connections between the nucleus accumbens (NAcc) and the insular, amygdala, and hippocampus and weaker functional connections within the DMN and reward circuits (24, 25). FC within the FPN was also weakened, while FC within the sensorimotor network was enhanced. These changes correlated with the severity of depressive symptoms (24). In particular, the strength of functional connections between OFC and other brain regions such as reward networks, SN and DMN plays an important role in predicting the severity of depressive symptoms (23). Meanwhile, for depression with insomnia, the surface area of the right insular lobe, left inferior frontal triangle, left frontal pole, right superior parietal lobule, right medial orbitofrontal cortex and right superiormarginal gyrus were found to be correlated with the severity of insomnia (26). FC also had characteristic changes, such as connections between the left thalamus and the left temporal pole, between the right medial superior frontal gyrus and the left insular lobe, and between the left and right orbitofrontal cortices in the DMN changed (27); enhanced connections between the amygdala and the supplementary motor area (SMA) and bilateral postcentral gyrus (POCG) in the SMN (28); and weakened connections between the septal nucleus of the SN and Crus I in the right cerebellum (29). Based on the high heterogeneity of chronic insomnia, it is important to explore the mechanism of insomnia comorbidity with depression.

Recently, Reimann et al. collected 39 structural and functional neuroimages to conduct a comprehensive analysis of brain structure and function changes in insomnia patients. The study found that the subgenual anterior cingulate cortex (sgACC) regions in insomnia patients showed significant abnormalities through activation likelihood estimation (ALE) algorithm (30). sgACC is the most ventral part of the anterior cingulate cortex (ACC), located deep in the cerebral cortex, and consists of two cellular structural regions s24 and s32, where s24 is involved in processing autobiographical negative or sad stimuli in the present or recalling the past, and s32 is associated with fear processing and rumination behavior, thus playing an important role in mood regulation, self-reflection and cognitive control (31, 32). Acute sleep deprivation or ID can significantly affect emotional processing and negatively affect overall emotional state, which may be related to sgACC dysfunction. These abnormalities in emotional processing and rumination observed in ID are strongly correlated with the role of sgACC in depression (MDD). In MDD, sgACC has been shown to play a key role in mood disorders, and recent multimodal meta-analysis and multicenter collaborative studies have also found that sgACC regions are altered in gray matter volume and functional connectivity to the amygdala in depressed patients (26). Importantly, sgACC is also the precision treatment target for Stanford Neuromodulation Therapy for treatment-resistant depression (33). In conclusion, sgACC plays an important role in sleep regulation and mood regulation, so it is necessary to further explore the sgACC network characteristics of CID patients with depression (34).

In this study, we sought to examine alterations in the sgACC functional network in CID patients, both with and without depressive symptom. First, group differences of sgACC-based functional connectivity were assessed across three groups: good sleep control (GSC), CID patients with depressive symptom (CID-D) and CID patients without depressive symptom (CID-nD). Second, the clinical relevance of altered sgACC network connectivity in CID patients was evaluated. Third, mediation analysis was applied to examine whether changes in the sgACC network mediate the relationship between insomnia severity and depressive symptoms in CID patients. Based on prior research on sgACC networks in major depressive disorder (MDD), we hypothesized that CID patients with comorbid depression would exhibit specific alterations in sgACC functional connectivity, and that these alterations would mediate the association between insomnia and depressive symptoms.

## 2 Materials and methods

### 2.1 Participants

A total of 210 participants were enrolled in the research, including 140 CID patients and 70 good sleep controls (GSC). All participants were sourced from the Neurology Department of the Chengdu Second People’s Hospital and were of Han descent and right-handed. the study received approval from the Institutional Ethics Committee of Chengdu Second People’s Hospital (Approval No.2020021), and written informed consent was obtained from each participant. During functional neuroimaging preprocessing, 13 participants (7 CID, 6 GSC) demonstrating excessive motion artifacts - defined as translational displacements surpassing 2.5 mm or rotational deviations exceeding 2.5° were excluded from subsequent analyses. The final analytical cohort comprised 133 CID patients and 64 HC participants.

Inclusion criteria for CID included: (1) meeting the diagnostic criteria for CID as per the International Classification of Sleep Disorders, 3rd Edition (1); (2) scoring above 7 on the Pittsburgh Sleep Quality Index (PSQI); (3) abstaining from hypnotic medications for at least 2 weeks prior to neuropsychological assessments and MRI scans; and (4) being aged between 18 and 60 years. The criteria for inclusion in the GSC group mirrored those for CID participants, with the exception of absence of sleep disturbances, PSQI score below 7. Exclusion criteria for all groups were consistent and encompassed: (1) presence of other neuropsychiatric disorders or severe chronic illnesses (e.g., diabetes, cardiovascular conditions, and cancer); (2) co-occurrence of other sleep disorders like sleep apnea, narcolepsy, circadian rhythm disruptions, and sleep movement disorders; (3) patients with severe depressive or anxiety symptoms (a cutoff ≥ 70 on the Self-Rating Anxiety Scale (SAS), or Self-Rating Depression Scale (SDS)) (35, 36) (4) history of substance abuse (e.g., drugs, nicotine, and alcohol); (5) contraindications for MRI; and (6) evidence of brain lesions or white matter hyperintensities on conventional T2-weighted MRI scans.

### 2.2 Assessment

All participants underwent comprehensive clinical and behavioral evaluations conducted by two seasoned neurologists who reached a consensus on all assessments. The PSQI (37, 38) was employed to evaluate the sleep quality of individuals with CID, while the SDS and SAS are used to assess depressive and anxiety symptoms in CID patients. For the CID cohort, participants were categorized into CID-D (SDS score ≥50) and CID-nD (SDS score <50) groups based on the dividing criterion of the China norm result (36, 39). All assessments were completed prior to the MRI scanning procedure to avoid potential confounding effects of scan-related anxiety on symptom ratings.

### 2.3 Image acquisition and preprocessing

MRI scans were conducted on all participants using a GE 3.0-TESLA scanner (GE Healthcare Discovery Pioneer, General Electric, Milwaukee, WI) at the Second People’s Hospital of Chengdu City between 4 p.m. and 6 p.m. High-resolution structural images were acquired through a gradient recall echo sequence with parameters: repetition time/echo time(TR/TE) = 7.06/3.04 ms; flip angle (FA) = 12°; acquisition matrix = 256 × 256; field of view (FOV)= 240 × 240 mm; thickness = 1.0 mm; gap=0; number of slices =192. Resting-state functional MRI data were obtained using an 8-minute echo planar imaging sequence with parameters: TR/TE = 2000/30 ms; FA = 90°; acquisition matrix = 64 × 64; slice thickness = 4 mm; inter-slice gap = 0.5 mm; and 33 interleaved transverse slices, resulting in 240 volumes over approximately 8 minutes. Participants were instructed to relax with eyes closed but not to sleep, and were provided with ear plugs and foam pads to minimize noise and head movement. Post-scan, participants confirmed their wakefulness during data acquisition.

Resting-state functional magnetic resonance imaging (fMRI) data were preprocessed with SPM12 (http://www.fil.ion.ucl.ac.uk/spm) and GRETNA 2.0 (https://www.nitrc.org/projects/gretna) in MATLAB R2022b (MathWorks, Inc.). Initially, the first ten volumes were excluded to address magnetization equilibrium and subject acclimatization to the experimental setting. Subsequently, slice timing and head motion corrections were applied to the remaining 230 images. All processed images underwent normalization to the Montreal Neurological Institute standard template space using the DARTEL algorithm, with resampling to 3×3mm³ voxels, detrending, and filtering (0.01-0.08 Hz). Regression analysis included removal of white matter, cerebrospinal fluid, and 24 covariates related to head motion and noise, while the global signal was retained due to its potential correlation with resting-state fMRI data. Data were smoothed by using a Gaussian kernel of 6mm. Participants who exhibited excessive head motion artifacts (head motion >1 mm relative to the first or in-frame displacement exceeding 2.5 mm or 2.5° angular motion) were excluded from the analysis.

### 2.4 sgACC-based FC analysis

The analysis of voxel functional connectivity (FC) in the subgenual anterior cingulate cortex (sgACC) (30) was conducted utilizing the Resting-State fMRI Data Analysis Toolkit (DPABI; http://rfmri.org/DPABI). Initially, a seed point was defined at MNI coordinates (x,y,z: 0, 34, -14) with a radius of 6mm to establish the functional network connections of the sgACC. Subsequently, a functional connectivity map was created by computing Pearson correlation coefficients between the mean time series of the seed region and all other voxels across the entire brain. Fisher’s z transformation was applied to enhance the correlation coefficients and approximate a normal distribution (40, 41). Ultimately, the FC matrix encompassing the entire brain for each participant was derived for subsequent analyses.

### 2.5 Statistical analysis

First, statistical analyses were conducted using SPSS 27.0 (IBM Corp., Armonk, NY, USA). A preliminary assessment of normality of the data distribution was performed by Kolmogorov-Smirnov method. For variables that did not conform to normal distribution, including demographic parameters (age, education level) and psychometric indicators (PSQI, SAS, SDS), Kruskal-Wallis H test was used for comparison between groups; clinical characteristics (disease duration) were evaluated by Mann-Whitney U test; chi-square test was used to test differences in categorical indicators (sex). Statistical significance was set at *p* < 0.05.

Second, The brain networks of the sgACC among the three groups of subjects were compared using Voxel-wise ANCOVA with the DPABI, with age, sex and years of education as covariates. Then, multiple comparison correction using a Gaussian random field (GRF correction) was performed with the significance threshold set at voxel *p* < 0. 05 and cluster *p* < 0. 05.

Third, Partial correlation analysis was used to explore the association between altered sgACC brain network and clinical features (SDS, SAS and PSQI scores) in the CID group after controlling for the effects of age, sex, duration of disease, and educational level, with significance set at *p* < 0.05 after multiple comparisons adjusted for false discovery rate (FDR).

Last, We conducted a causal mediation analysis within the counterfactual framework using the mediation package in R to examine whether the altered sgACC FC network mediated the relationship between PSQI and SDS in CID group. The analysis adjusted for covariates (e.g., age, gender, education) and employed nonparametric bootstrapping (1,000 simulations)to estimate 95% confidence intervals for the effects. Key estimands included the average causal mediation effect (ACME), representing the indirect effect of PSQI on SDS through sgACC FC network; the average direct effect (ADE), capturing the residual effect of PSQI on SDS independent of the mediator; and the total effect, summing direct and indirect pathways. To assess robustness to unmeasured confounding, we performed a sensitivity analysis by varying the correlation strength (ρ) between hypothetical confounders and the mediator/outcome, determining the threshold at which ACME would be nullified (*ρ* = 0.25). This approach quantified how much unmeasured confounding would be required to invalidate the mediation effect, expressed via variance explained (R²) in the mediator and outcome.

## 3 Results

### 3.1 Demographic and clinical features

As shown in **Table 1**, no significant differences in sex or education years were observed between the CID subgroups and GSC group. For age, the CID-nD group was significantly older than the CID-D group (p=0.041), while the GSC group did not differ in age from either CID subgroup; age was therefore included as a covariate in subsequent analyses. Significant differences in PSQI, SDS, and SAS scores emerged between the combined CID group and GSC group (*p* < 0.001), and between CID subgroups also differed significantly in SDS and SAS scores (*p* < 0.001). Within the CID group, disease duration showed no significant correlations with PSQI, SDS, or SAS scores (*p* > 0.05), but PSQI was positively correlated with SDS (*r* = 0.274, *p* = 0.001), and SDS scores were strongly positively correlated with SAS scores (*r* = 0.82, *p* < 0.001).

**Table 1:** Demographic and clinical characteristics of the participants.

Variables	GSCs (n=64)	CID-nD (n=66)	CID-D (n=67)	P-value
Age	34.70±10.35	37.95±11.85	33.16±10.25	<0.05
Sex (male)	23	21	15	0.222
Education year	15.02±3.52	15.15±3.26	15.49±2.37	0.504
PSQI score	3.77±4.57	12.29±3.17	13.55±2.93	<0.001
SDS score	34.58±7.00	41.73±5.63	59.67±8.46	<0.001
SAS score	32.95±6.70	41.73±5.63	54.81±11.30	<0.001
Duration of illness (yesr)	/	7.058±5.00	6.04±6.44	0.372

CID-nD, chronic insomnia disorder without depression group; CID-D, insomnia with depression group; GSC, good sleep controls; PSQI, Pittsburgh Sleep Quality Index; SDS, Self-Rating Depression Scale; SAS, Self-Rating Anxiety Scale.

### 3.2 Group differences in the sgACC-FC network

**Figure 1:**
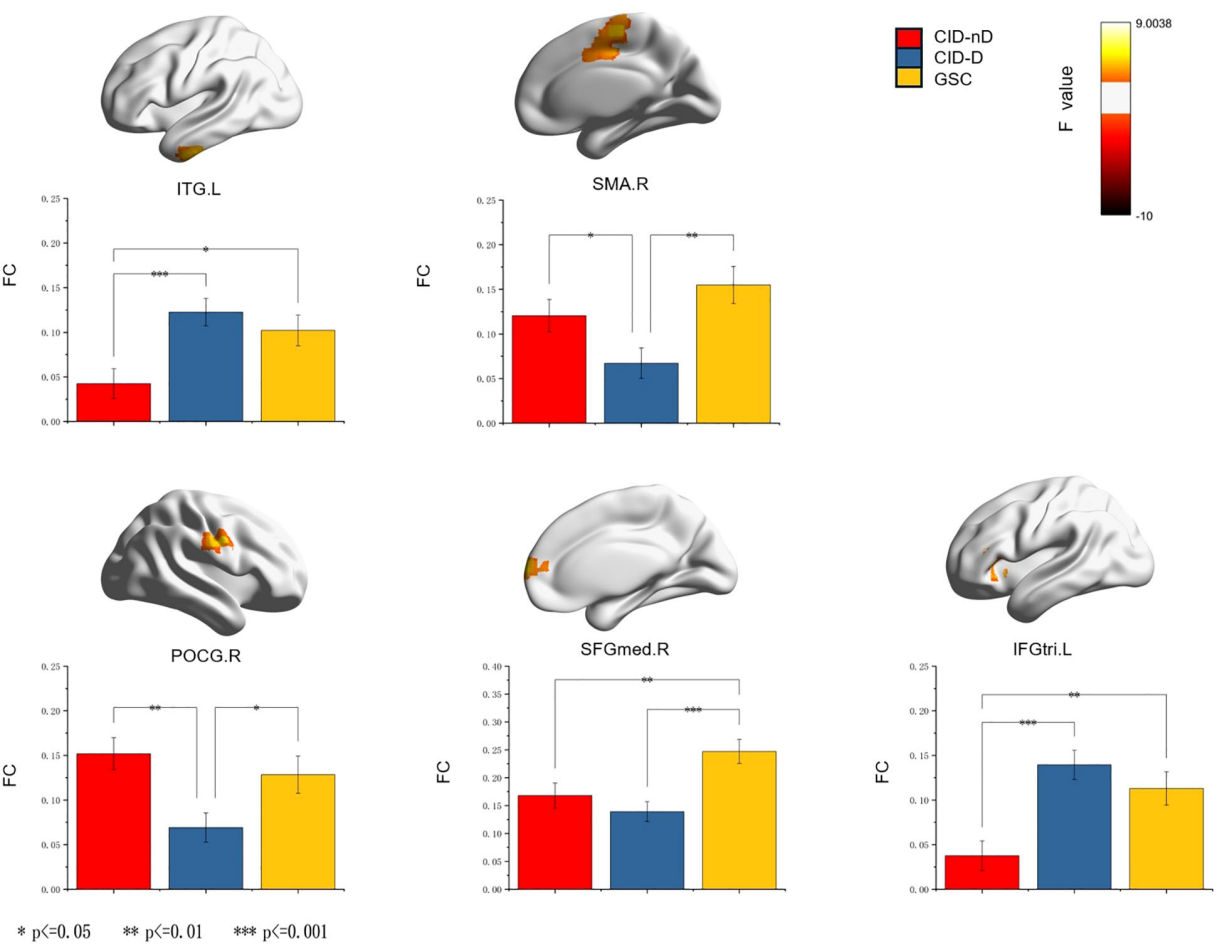
The sgACC FC network differences among three groups (voxel *p* < 0.05, cluster *p* < 0.05, GRF corrected). Asterisks indicate significant differences in *post hoc* analyses corrected by the LSD method (*p < 0.05, **p < 0.01, ***p < 0.001). sgACC, subgenual anterior cingulate cortex; FC, functional connectivity; ITG.L, left inferior temporal gyrus; SMA.R, right supplementary motor area; POCG.R, right postcentral gyrus; SFGmed.R, right medial superior frontal gyrus; IFGtri.L, left inferior frontal gyrus, triangular part.

Voxel analysis of variance (ANOVA) showed significant differences in sgACC FC among the three groups in five brain regions: including the left inferior temporal gyrus (ITG), inferior frontal gyrus (IFGtri), right supplementary motor area (SMA), posterior central gyrus (POCG) and medial superior frontal gyrus (SFG). These findings are summarized in **Table 2**; **Figure 1**. A *post hoc* analysis of FC values in three groups of brain regions showed that the two CID groups showed reduced FC in SFGmed.R compared with the GSC. Compared to CID-nD, CID-D patients showed increased sgACC FC on ITG.L and IFGtri.L, while they showed decreased sgACC FC in POCG.R and SMA.R.

**Table 2:** The time ×group effect on the sgACC functional network.

Brain region	Boadmann’s area	Cluster size	MNI coordinates(RAI)			Peak *F* value
			x	y	z	
ITG.L	20	58	-58	-18	-27	6.58
SMA.R	6	188	-9	-6	48	7.51
POCG.R	3	53	57	-3	39	5.96
SFGmed.R	10	61	24	51	18	6.98
IFGtri.L	45	54	-42	21	0	9.00

sgACC, subgenual anterior cingulate cortex; FC, functional connectivity; ITG.L, left inferior temporal gyrus; POCG.R, right postcentral gyrus; IFGtri.L, left inferior frontal gyrus, triangular part; SMA.R, right supplementary motor area; SFGmed.R, right medial superior frontal gyrus.

### 3.3 Clinical significance of altered sgACC FC in the CID group

**Figure 2:**
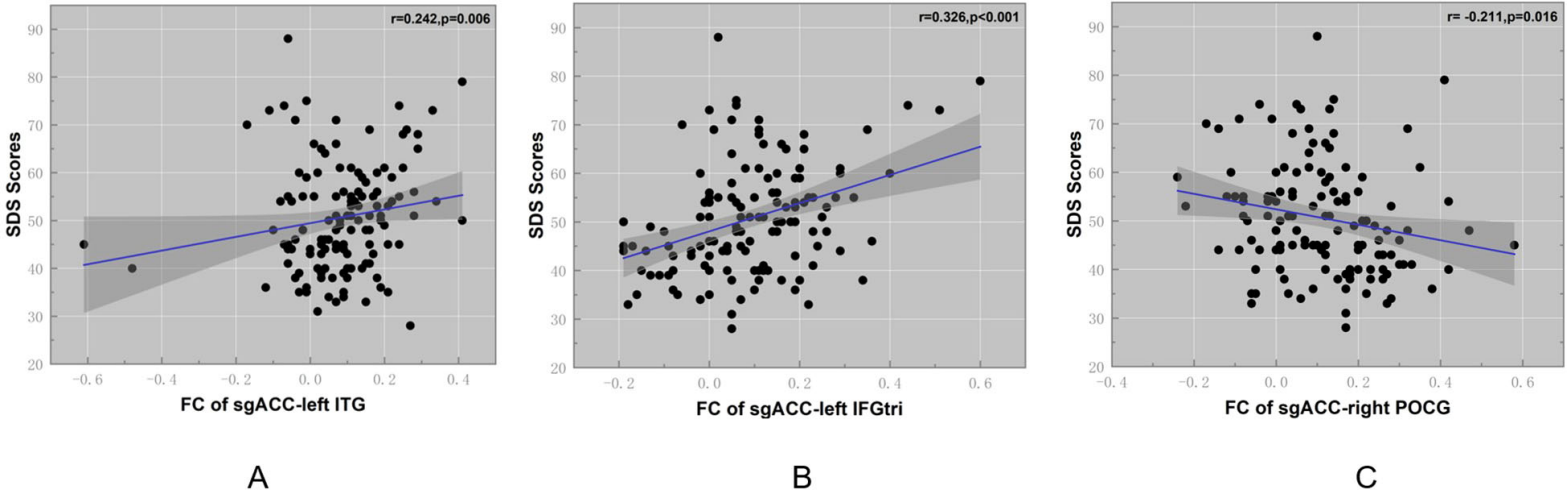
Clinical significance of altered sgACC FC in patients with chronic insomnia. **(A)** Correlation analysis results of the left inferior temporal gyrus after controlling for age, gender, and disease duration. **(B)** Correlation analysis results of the triangular part of the left inferior frontal gyrus after controlling for age, gender, and disease duration. **(C)** Correlation analysis results of the right postcentral gyrus after controlling for age, gender, and disease duration. sgACC, subgenual anterior cingulate cortex; FC, functional connectivity; ITG, inferior temporal gyrus; POCG, postcentral gyrus; IFGtri, triangular part of the inferior frontal gyrus; SDS, Self-Rating Depression Scale.

Partial correlation analysis was used to explore the relationship between abnormal sgACC FC and clinical symptoms in CID group. After controlling for the effects of sex, age, duration of disease and education level, the altered sgACC FC in ITG.L was positively associated with SDS score in CID patients (**Figure 2A**, r = 0.24, *p* = 0.006), the FC between sgACC and IFGtri.L was positively correlated with SDS scores (**Figure 2B**, r = 0.32, *p* < 0.001), while the FC between sgACC and POCG.R was significantly negatively correlated with SDS scores in patients with CID (**Figure 2C**, r = -0.211, p=0.016). No significant correlation between abnormal sgACC FC and SDS or SAS was found in the CID group (*p* > 0.05).

### 3.4 The mediating role of sgACC network in the relationship between insomnia and depressive symptoms in CID group

**Figure 3:**
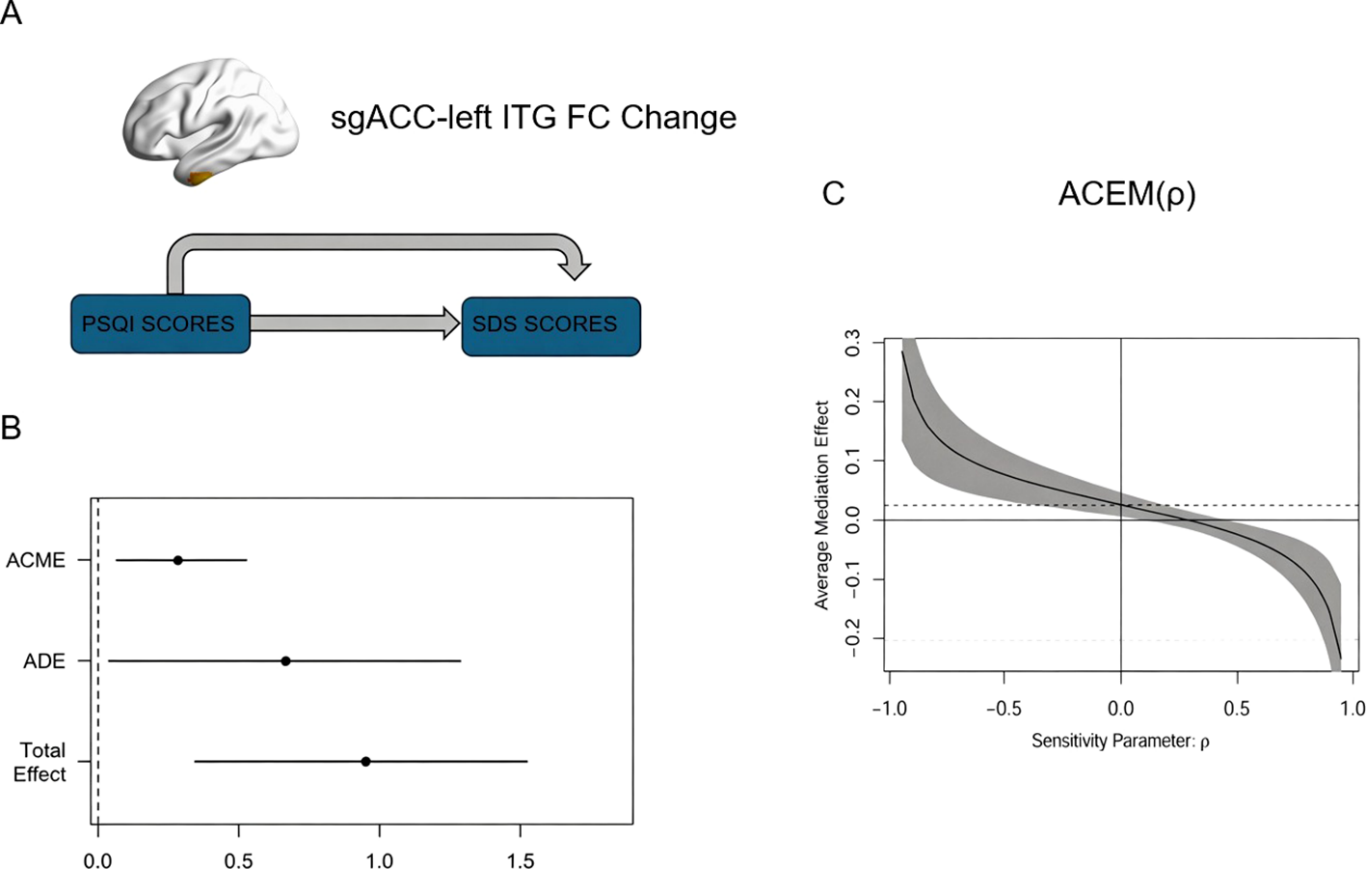
Mediation analysis results. **(A)** Conceptual model depicting the relationships between sgACC and ITG.L functional connectivity change, SDS scores, and PSQI Scores. The arrows indicate the direct and indirect pathways through which PSQI Scores influence SDS Scores. **(B)** Estimated effects from the mediation analysis. The ACME and ADE are shown with their confidence intervals, along with the Total Effect of ITG.L functional connectivity changes on SDS Scores. **(C)** Sensitivity analysis of the ACME, illustrating how the mediation effect varies with changes in the sensitivity parameter ρ. The shaded area represents the confidence interval of the mediation effect across different values of ρ. PSQI, Pittsburgh Sleep Quality Index; ITG.L, left inferior temporal gyrus; sgACC, subgenual anterior cingulate cortex; FC, functional connectivity; SDS Scores, Self-Rating Depression Scale scores; ACME, Average Causal Mediation Effect; ADE, Average Direct Effect.

As shown in **Figure 3**; **Table 3**, the mediation analysis revealed a significant indirect effect of sleep quality on depressive symptoms through sgACC-ITG.L FC (ACME = 0.28, 95% CI [0.07, 0.54], *p* = 0.01), accounting for 29.9% of the total effect (total effect = 0.95, *p* = 0.002). The direct effect of PSQI on SDS remained significant (ADE = 0.67, 95% CI [0.02, 1.35], *p* = 0.04), suggesting additional pathways beyond the mediator. Sensitivity analysis indicated that the mediation results were robust to unmeasured confounding unless hypothetical confounders explained 6.25% of the variance in both sgACC-ITG.L FC and SDS or had a correlation strength (ρ) exceeding 0.25 with both variables. This implies that the observed mediation effect would only be negated by strong, unaccounted confounders—beyond the influence of typical covariates like age or gender. The findings support the partial mediating role of the sgACC FC network in the sleep-depression link. No significant mediation effect was found in other altered sgACC FC network in CID group.

**Table 3:** Mediation analysis of altered sgACC-left ITG FC as a Mediator in PSQI scores induced SDS scores changes.

Variables	Estimate	95%CI lower	95%CI upper	P-value
ACME	0.28	0.07	0.54	0.014
ADE	0.67	0.02	1.35	0.044
Total Effect	0.95	0.29	1.57	0.002
Prop.Mediated	0.29	0.06	0.95	0.016

PSQI Scores, Pittsburgh Sleep Quality Index scores; ITG.L, left inferior temporal gyrus; sgACC, subgenual anterior cingulate cortex; FC, functional connectivity; SDS Scores, Self-Rating Depression Scale scores; ACME, average causal mediation effect; ADE, average direct effects.

## 4 Discussion

The present study investigates specific alterations in the sgACC FC network in patients with CID, with and without depressive symptoms. Our findings reveal that CID patients exhibit significantly reduced FC between sgACC and SFGmed.R and SMA.R compared to GSC. In addition, CID-D patients demonstrate notably decreased FC between sgACC and the SMA.R and POCG.R, and increased FC between sgACC and ITG.L and IFGtri.L compared to CID-nD. The altered sgACC FC shows significant correlation with the SDS in CID group. Furthermore, the FC between sgACC and ITG.L partially mediates the relationship between insomnia and depression symptoms. These results illuminate the neural circuitry underlying the insomnia-depression comorbidity and pinpoint the sgACC network as a promising target for developing precise neurobiological interventions for CID patients with depression.

Our study revealed sgACC functional network alteration in CID patients compared to GSC. Specifically, a decrease in FC was observed in SFGmed.R. The DMN encompasses the SFGmed.R, which is notably active during rest and plays a crucial role in self-referential processes, introspection, episodic memory, emotional regulation, and various cognitive functions (42). The reduced connectivity between sgACC and DMN regions is linked to the underlying pathophysiology of CID (43). For instance, Gong’s research indicates that diminished DMN functionality in CID patients correlates with heightened nocturnal arousal and pre-sleep rumination (44), which aligns with our results. The sgACC, situated at the ventral aspect of the anterior cingulate cortex (ACC) near the brain’s core, is implicated in emotional modulation and cognitive processing (30, 45). The diminished FC between sgACC and SFGmed.R may signify a weakened regulatory influence of the sgACC on processing self-relevant emotional information. This impairment could contribute to the inability of individuals with insomnia to inhibit excessive attention and negative anticipations related to sleep onset, thereby exacerbating their sleep disturbances.

The present study found CID patients with comorbid depression exhibit specific alterations in sgACC FC network. The CID-D group showed decreased sgACC FC in POCG.R and SMA.R. They are the crucial regions in the SMN, responsible for receiving and integrating somatosensory input. It exhibits extensive connections with the hippocampus, amygdala, and prefrontal cortex, contributing significantly to emotional regulation and cognitive processes (46, 47). Symptoms commonly observed in individuals with depression include persistent low mood, diminished interest, and cognitive impairments (48). The disrupted connectivity observed in CID-D patients may indicate compromised regulation of the sensory cortex in individuals with insomnia, leading to delays in sensory processing, potentially contributing to emotional blunting in depression. Our discovery of a negative correlation between the FC of sgACC-POCG.R and the severity of depression lends support to this hypothesis. This finding aligns with Zhu’s prior research, which highlighted that weakened connections between the visual network and SMN impact symptoms in individuals with depression (49, 50). Furthermore, the increased FC between the sgACC and POCG.R in CID-nD patients compared to individuals with GSC may indicate sensory processing abnormalities in those with chronic insomnia alone. This heightened connectivity could render individuals more sensitive to their sleep environment, thereby increasing emotional distress and susceptibility to developing depressive symptoms. Additionally, this altered FC pattern may serve as a potential biomarker for distinguishing between individuals with insomnia and those with depression. Recent studies have found that the SMA is associated with psychomotor retardation, and repetitive transcranial magnetic stimulation over the SMA has been confirmed to reduce psychomotor retardation in patients with MDD (51). Therefore, our findings that the decreased FC between sgACC and SMA.R may be related to this symptom. The reduction of this connectivity may also be linked to sleep. Previous studies have indicated that the SMN remains partially active during rest to maintain motor inhibition during rapid eye movement (REM) sleep, thereby ensuring the continuity of sleep (52, 53). Therefore, a decrease in FC may impair the regulation of rapid eye movement inhibition, subsequently affecting the sleep-wake cycle, and leading to sleep disorders. However, research by Cheng suggests that enhanced connectivity in the SMN could potentially lead to sleep disturbances (54). Consequently, it is necessary to conduct further studies to explore and clarify the association between these changes in functional connectivity and chronic insomnia.

Our findings reveal that FC between sgACC and ITG.L was heightened in CID-D compared to CID-nD. ITG.L is associated with the cognitive control network and limbic system, playing a key role in auditory processing, language comprehension, emotional processing, and memory retrieval (55). Previous research has suggested that depressed individuals exhibit enhanced negative emotional memory retrieval and diminished cognitive control (56, 57). The increased FC between sgACC and ITG.L in the CID-D group may signify a facilitation of negative emotional memory activation and retrieval. Furthermore, correlation analyses demonstrated a positive relationship between the FC of sgACC-ITG.L and the severity of depression, providing further support for this interpretation. IFGtri.L plays a crucial role within the frontoparietal network and central executive network, which are implicated in language processing, working memory, and cognitive control (58). The sgACC is closely associated with emotion regulation, particularly in fear perception and rumination (30). Elevated FC between sgACC and IFGtri.L in CID-D individuals may reflect altered neural processing at the interface of cognitive control and emotional regulation, though its functional relevance remains unclear. The positive correlation between sgACC-IFGtri.L FC and depression severity suggests that this altered connectivity may be associated with the maintenance of depressive symptoms, but this interpretation remains speculative and requires further research.

Our study found that in the association pathway between sleep and depression in patients with CID, only the ITG.L exerted a significant mediating effect. The core reason lies in the unique positioning of ITG.L in emotional memory processing and the neural circuit of sleep-emotion interaction. As a core node in emotional memory processing, the ITG.L is specifically involved in the encoding, consolidation, and retrieval of negative emotional memories (55). Sleep disturbances disrupt the normal consolidation of emotional memories, leading to over consolidation of negative memories and difficulty in their elimination (59, 60). Moreover, the enhanced FC between sgACC and ITG.L facilitates the retrieval of negative memories during wakefulness, strengthens their encoding and retention (61, 62), and forms a vicious cycle of “poor sleep → negative memory recall → low mood → poorer sleep (63). Ultimately promoting the occurrence and development of depression. This mediating pathway is consistent with the “emotional memory over consolidation theory” of depression, highlighting the crucial mediating role of ITG.L in the pathological process of comorbid sleep disturbance and depression (64). Although there was a positive correlation between sgACC-IFGtri.L and SDS scores, no mediating effect was observed. This may be attributed to the primary function of IFGtri.L in cognitive control (65), which focuses more on the “*post-hoc* regulation” of emotional responses rather than the initial encoding and consolidation of emotional information, thus lacking a mediating effect. Similarly, despite the negative correlation between sgACC-POCG.R and SDS scores, no mediating effect was found. This may be because POCG.R, as a key region in the sensorimotor network, is mainly involved in somatosensory processing (46). The reduced functional connectivity in POCG.R may indicate “impaired emotion-sensory integration function”, which is a characteristic of depressive symptoms rather than serving as a “causal intermediary node” linking sleep and depression.

There are limitations in this study. First, the sample size is small and it is a cross-sectional design. In the future, it is necessary to expand the sample and carry out a multicenter study to verify the results and explore whether sgACC-POCG.R FC can be used as a potential marker to distinguish CID-nD complicated with depression. Second, although age was controlled as a covariate, prior studies have established that age is tightly linked to spontaneous brain activity and functional connectivity patterns. Thus, age imbalance between CID subgroups may confound the results, which could be mitigated by stricter age matching or age stratification in future studies. Third, this study included patients with insomnia comorbid with depression, but lacked a patient group with depression comorbid with insomnia. Whether there are common sgACC network abnormalities between the two groups needs further validation.

## 5 Conclusions

The present study demonstrates that comorbid depression in CID is associated with distinct alterations in the sgACC functional network, includes hyperconnectivity of the sgACC with limbic and cognitive control network (ITG.L, IFGtri.L) and hypoconnectivity with sensorimotor network (SMA.R, POCG.R), which correlate with depressive symptom severity. Crucially, the sgACC-ITG.L pathway was identified as a significant mediator between poor sleep quality and depression. These results illuminate the neural circuitry underlying the insomnia-depression comorbidity and pinpoint the sgACC network as a promising target for developing precise neurobiological interventions for CID patients with depression.

## Data Availability

The original contributions presented in the study are included in the article/supplementary material. Further inquiries can be directed to the corresponding authors.
